# COVID-19: complexity of disease severity revealed by systemic and localized single cell immune atlas

**DOI:** 10.1038/s41392-021-00587-3

**Published:** 2021-04-16

**Authors:** Nehad M. Alajez

**Affiliations:** 1 Translational Cancer and Immunity Center (TCIC), Qatar Biomedical Research Institute (QBRI), Hamad Bin Khalifa University (HBKU), Qatar Foundation (QF), Doha, Qatar; 2 College of Health & Life Sciences, Hamad Bin Khalifa University (HBKU), Qatar Foundation (QF), Doha, Qatar

**Keywords:** Infectious diseases, Infection

A recent study published in Cell by Ren et al. revealed the immune features associated with age, gender, severity, and stage of Coronavirus Disease 2019 (COVID-19) employing single-cell RNA-sequencing.^1^ The findings highlighted a role for megakaryocytes and monocytes in the circulation as potential sources of cytokine storms oftentimes seen in severe COVID-19 and suggested plausible crosstalk between inflammatory cells in the lungs and the periphery.

As the COVID-19 global pandemic surpass 130 million confirmed cases and over 2.9 million deaths thus far, increasing efforts are being exerted to understand the etiology and severity of this disease with the aim to develop effective preventive and therapeutic strategies. Severe acute respiratory syndrome coronavirus 2 (SARS-CoV-2) infection does not affect all individuals in the same way, whereas the majority of COVID-19 patients experience mild to moderate symptoms, significant proportion of the patients exhibit severe symptoms, including acute respiratory distress syndrome (ARDS), and ultimately could succumb to death. Therefore, factors, in particular immune components, pertaining to disease severity and the origin of cells provoking inflammatory cytokine storms in severe COVID-19 patients are currently being unraveled employing cutting-edge approaches such as single-cell RNA-sequencing (scRNA-seq) of such factors in bronchoalveolar lavage fluid (BALF) and in the circulation. Ren et al. employed scRNA-seq and characterized the immune and cell landscape consisting of approximately 1.4 million cells in 284 samples derived from 196 COVID-19 patients (mild/moderate *n* = 22, severe *n* = 54, and convalescent patients *n* = 95) as well as in healthy controls (*n* = 25).^1^ Majority of the samples were from peripheral blood mononuclear cells (PBMCs, *n* = 249), while the rest were from BALF, sputum, or pleural fluid mononuclear cells. Global overview revealed enrichment of proliferating CD4+ and CD8+ T cells, and plasma cells in BALF compared to PBMCs, while proliferative and activated T and B cells as well as macrophages were more abundant in patients with severe COVID-19 during progression stage. Age and gender were also found to influence repertoire diversity of B cell receptor (BCR) and T cell receptor (TCR). Naive CD8+ T cells (T_CD8_c01 − LEF1) exhibited highest association with patient age, while T_CD4_c04 − ANXA2, T_CD4_c08−GZMK − FOS^high^, and T_CD8_c02 − GPR183 were more linked to gender.

**Fig. 1:**
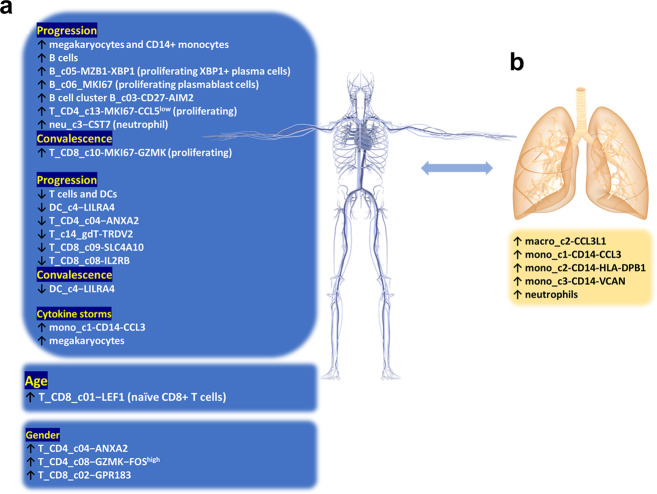
Schematic presentation of altered immune subsets in the periphery and lungs from COVID-19 patients. Frequencies of immune subsets in the circulation (**a**) and lung tissue (**b**) from patients with COVID-19. ↑ indicates increased while ↓ indicates decreased frequencies

In the periphery, the authors reported enrichment of megakaryocytes and CD14+ monocytes during the progression stage of patients with severe COVID-19 (Fig. 1a). Notably, proliferating XBP1+ plasma cells, and Neu_c3 − CST7 neutrophil cluster, were associated with COVID-19 severity, with the later also being linked to age and disease stage. B cell cluster B_c03-CD27-AIM2 was also enriched during progression stage of severe COVID-19, while proliferating plasmablast cells (B_c06_MKI67) were elevated in the circulation of severe COVID-19 patients. Looking into correlation between T cell clusters and COVID-19 disease severity, the authors reported enrichment of the T_CD4_c13-MKI67-CCL5^low^ cluster primarily in the disease progression stage of patients with severe COVID-19. In the context of cytotoxic T cells, the T_CD8_c10-MKI67-GZMK proliferative effector memory CD8+ T cell cluster was also enriched in severe COVID-19 patients, primarily during recovery. Interestingly, plasmacytoid dendritic cell cluster DC_c4 − LILRA4, T_CD4_c04 − ANXA2, T_c14_gdT-TRDV2 (γδ T cells), T_CD8_c09-SLC4A10, and T_CD8_c08-IL2RB clusters were decreased in the progression stage of severe COVID-19 patients (Fig. 1a). Despite enrichment of various B and T cell clusters in sever COVID-19 patients, the authors reported lack of common BCRs or TCRs among patients, interestingly however, the BCR repertoire of COVID-19 patients exhibited skewed VDJ rearrangement (including IGHV3 and IGHV1) compared with those seen in healthy controls.

The exact sources of inflammatory cytokine storms in the context of COVID-19 disease severity remains controversial. Herein, the authors pinpointed Mono_c1-CD14-CCL3 and megakaryocytes as potential sources of cytokine storms in PBMCs. In agreement with those data, Mono_c1-CD14-CCL3 cells expressed elevated levels of CCL3, TNF, and IL1RN, which were also present in the plasma of patients with severe COVID-19. While in BALF, the authors identified several hyper-inflammatory cell subtypes, including Macro_c2-CCL3L1, Mono_c1-CD14-CCL3, Mono_c2-CD14-HLA-DPB1, and Mono_c3-CD14-VCAN clusters and neutrophils, suggesting those immune subsets as potential sources provoking localized inflammatory response in the lungs (Fig. 1b), which in part is consistent with Zhang et al. who reported the presence of CD14+ in severe COVID-19.^2^

The authors subsequently sought to detect SARS-CoV-2 in various cell subsets which revealed the presence of SARS-CoV-2 viral RNAs in various epithelial cells (squamous, ciliated, and secretory) as well as in several immune subsets, including macrophages, neutrophils, plasma and T cells, and NK cells, which correlated with the expression of interferon-stimulated genes (ISGs) in the same cell subsets. A remaining question however is, how immune cells acquire viral RNA given their lack of expression of ACE2 and TMPRSS2, which are essential for SARS-CoV-2 entry.

Data reported by Ren and colleagues provide global view of the immune cell portrait in the circulation and respiratory system of a large cohort of COVID-19 patients compared to healthy controls and highlighted the heterogeneity of local and peripheral immune responses, and suggested plausible crosstalk between inflammatory cells in the lungs and the periphery. While different scRNA-seq studies have reported different immune portraits in their respective COVID-19 cohorts, the presence of inflammatory innate immune cells appears as a hallmark for severe COVID-19. Findings by Ren and colleagues are consistent with our recent publication implicating inflammatory macrophages and neutrophils in severe COVID-19.^3^ During the disease progression stage of severe COVID-19 patients, Ren et al. reported S100A9 and S100A8 to be significantly up-regulated in the majority of cell clusters in PBMCs and BALF. Those data are also in agreement with our recent finding where we reported S100A8 as a marker of neutrophils in BAL from severe COVID-19 patients,^3^ while expression of S100A9 in PBMCs from COVID-19 patients was reported by Wilk et al.^4^ Nonetheless, we also reported CCL3 as key marker associated with inflammatory macrophages from COVID-19 BALF, which would be consistent with the Macro_c2-CCL3L1 cluster reported by Ren et al. The authors also reported viral RNA-positive squamous cells to interact with macrophages and neutrophils in severe COVID-19 patients utilizing the S100A9/A8-TLR4 and ANXA1-FPR1 axes, which could provide novel axis for therapeutic intervention for severe COVID-19.

It becomes unequivocal that inflammatory immune cells are key players in cytokine storms, oftentimes seen in severe COVID-19 patients. However, a remaining question is why do severe COVID-19 patients develop such skewed immune response and whether ethnicity and genetic predisposition play a factor? Is this altered immune response the cause or the consequence of COVID-19 severity? With the emergence of new SARS-CoV-2 variants, it remains to be determined if similar patterns of immune responses are seen in the context of COVID-19 disease severity. How such findings could affect the way COVID-19 patients are currently being stratified and treated in the clinic remains to be addressed. Therapeutic and vaccination approaches aiming at enhancing anti-viral immunity, while inhibiting local and systemic inflammation might pave the way for more effective off-the-shelf and tailored treatment options for COVID-19.
